# Outcomes With Pulsed Field Ablation Versus Conventional Thermal Ablation for Paroxysmal Atrial Fibrillation: A Meta‐Analysis

**DOI:** 10.1002/joa3.70207

**Published:** 2025-10-22

**Authors:** Ahmed Elmorsy Mohamed, Ahmed Farid Gadelmawla, Zeyad Kholeif, Mohamed Elnady, Ahmed Diaa, Rana Rashwan, Ameer Awashra, Aya Elalfy, Imad Tahhan, Mohab Elnashar, Ranem Afify, Eman Mohyeldin, Haytham A. M. Dwaik, Jeanwoo Yoo, Islam Y. Elgendy

**Affiliations:** ^1^ NYU Langone Health New York New York USA; ^2^ Faculty of Medicine Menoufia University Menoufia Egypt; ^3^ Medical Research Group of Egypt Negida Academy Arlington Massachusetts USA; ^4^ Department of Internal Medicine Baptist Hospitals of Southeast Texas Beaumont Texas USA; ^5^ Faculty of Medicine Kafrelsheikh University Kafr El‐Sheikh Egypt; ^6^ Faculty of Medicine Al‐Azhar University Cairo Egypt; ^7^ Department of Internal Medicine NYC Health and Hospitals/Lincoln Bronx New York USA; ^8^ Department of Medicine An Najah National University Nablus Palestine; ^9^ Department of Internal Medicine Mayo Clinic Jacksonville Florida USA; ^10^ Department of Internal Medicine Wellington Regional Medical Center Wellington Florida USA; ^11^ Department of Internal Medicine University of Arkansas for Medical Sciences Little Rock Arkansas USA; ^12^ Faculty of Medicine Tanta University Tanta Egypt; ^13^ Faculty of Medicine University of Jordan Amman Jordan; ^14^ Department of Cardiovascular Medicine Mayo Clinic Jacksonville Florida USA; ^15^ Division of Cardiovascular Medicine Gill Heart and Vascular Institute, University of Kentucky Lexington Kentucky USA

**Keywords:** arrhythmia, cryoballoon ablation, PFA

## Abstract

**Background:**

Pulmonary vein isolation is a standard therapy for paroxysmal atrial fibrillation (AF). Pulsed field ablation (PFA) has emerged as a novel approach aiming to improve efficacy and safety over conventional thermal ablation (CTA) (radiofrequency and cryoballoon). This meta‐analysis evaluated outcomes of PFA versus thermal ablation in paroxysmal AF.

**Methods:**

Electronic databases were searched through May 2025 for randomized controlled trials (RCTs) and observational studies that compared the efficacy and safety of PFA versus CTA. The primary outcome was AF recurrence. Summary estimates were conducted using random effects.

**Results:**

A total of six studies, involving 1928 patients, were included. The incidence of AF recurrence was significantly lower among patients treated with PFA (risk ratio [RR] 0.67; 95% confidence interval [CI] 0.53–0.85). PFA was associated with a lower incidence of any atrial arrhythmia recurrence (RR 0.78, 95% CI: 0.61–0.99). The total procedure duration was significantly shorter with PFA (mean difference −21.46 min (95% CI: −26.04 to −16.88)), but there was no difference in fluoroscopy time. The rates of esophageal injury and phrenic nerve palsy were lower with PFA. However, the data were limited for these two outcomes, and a meta‐analysis was not conducted for them. There was no difference between the two groups in the incidence of stroke or pericardial tamponade.

**Conclusion:**

Among patients with paroxysmal AF undergoing catheter ablation, PFA is associated with favorable outcomes, including lower recurrence and shorter procedure time compared to conventional ablation modalities.

## Graphical abstract

Pulsed field ablation reduced atrial fibrillation recurrence and shortened procedure duration compared with conventional thermal ablation, with comparable safety outcomes. These findings support PFA as a promising alternative strategy for rhythm control in paroxysmal atrial fibrillation.

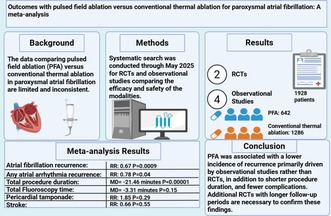

## 1 Introduction

Atrial fibrillation (AF) is the most prevalent tachyarrhythmia and is linked with worse outcomes, quality of life, and increased mortality [1]. Thermal ablation for pulmonary vein isolation (PVI) using radiofrequency ablation (RFA) and cryoballoon ablation (CBA) techniques has been an established management strategy for paroxysmal AF [2, 3, 4]. Nevertheless, atrial tachyarrhythmia frequently recurs, up to 18.4% in patients treated with RFA and 21.5% in patients treated with CBA. Other less common complications include phrenic nerve injury and atrio‐esophageal fistula [5, 6].

Novel techniques, such as pulsed‐field ablation (PFA), apply high‐voltage electrical pulses lasting microseconds to selectively target heart tissue, thereby minimizing harm to non‐myocardial structures. This approach offers consistent tissue ablation with a lower risk of complications [7, 8, 9].

Previous randomized controlled trials (RCTs) and observational studies showed inconsistent results regarding the outcomes of PFA in comparison with conventional thermal ablation (CTA) (RF and CBA) in paroxysmal AF. Recent RCTs, including Reddy et al. [10] and Reichlen et al. [11] were designed as non‐inferiority trials demonstrating that PFA is at least as effective as CTA in terms of AF and any atrial arrhythmia recurrence with better safety outcomes, while observational studies as Rocca et al. [12] suggested a possibly superior benefit of PFA, especially regarding recurrence rates. Moreover, several meta‐analyses have compared the two approaches, but none have specifically focused on the paroxysmal AF subtype. This meta‐analysis aims to evaluate the efficacy and safety endpoints of PFA and other CTA techniques in paroxysmal AF.

## 2 Methods

This meta‐analysis was conducted following the Preferred Reporting Items for Systematic Reviews and Meta‐Analyses (PRISMA) guidelines (Table S1) [13]. It was registered in the International Prospective Register of Systematic Reviews PROSPERO (Record number: CRD420251075168).

### 2.1 Data Source and Search Strategy

We searched Cochrane Library, Scopus, PubMed, and Web of Science from inception until May 2025 with the following search terms: (“pulsed‐field ablation”) AND ((“thermal ablation”) OR (“radiofrequency ablation”) OR (“cryoballoon ablation”)) AND (“Paroxysmal atrial fibrillation” OR “Paroxysmal AF”). The complete search strategy is provided in (Table S2). Duplicates were removed using EndNote (*Clarivate Analytics, PA, USA*). The retrieved references were screened in two steps: the first step was to screen titles/abstracts of all identified articles independently by two authors (R.A. and R.R.). The Rayyan website was used in this process. The second step was to screen the full‐text articles of the identified abstracts for final eligibility for meta‐analysis. Any disagreement during the screening processes was resolved by discussion and consensus with A.E.M.

### 2.2 Eligibility Criteria

We included RCTs and observational studies comparing PFA with CTA (i.e., RFA or CBA) and reported clinical outcomes for patients with paroxysmal AF. We excluded studies that did not report clinical outcomes or that had patients with types of AF other than paroxysmal AF, as we aimed to specifically examine this patient subgroup, given the limited availability of studies and reviews evaluating the efficacy and safety of PFA within this population. We also excluded non‐English studies and conference abstracts.

### 2.3 Data Extraction

Four authors (M.E., A.D., E.M., and H.D.) independently extracted baseline demographic characteristics and outcomes. Disagreement was resolved in a panel discussion with a third author (A.E.M.).

### 2.4 Outcomes

The primary outcome was AF recurrence (defined by the first occurrence of AF lasting at least 30 s, between 90 and 365 days after the ablation procedure). The secondary outcomes included: any atrial arrhythmia recurrence (defined by an episode of AF, atrial flutter, or atrial tachycardia lasting at least 30 s, between 90 and 365 days after the ablation procedure), total procedure time, total fluoroscopy time, and safety outcomes such as phrenic nerve palsy, esophageal injury, pericardial tamponade, stroke, and transient ischemic attack (TIA). Clinical outcomes were reported using an intention‐to‐treat basis at the longest reported follow‐up period.

### 2.5 Quality Assessment

We used the revised Cochrane risk‐of‐bias tool for RCTs (ROB2) [14] to evaluate the risk of bias in RCTs. This evaluation included an assessment of the randomization process, concealment of the allocation sequence, deviations from the intended interventions, utilization of appropriate analysis to estimate the effect of assignment to intervention, measurement of the outcomes, selection of the reported results, and overall risk of bias. The assessment of the methodological quality of the studies was classified as either low risk, with some concerns, or high risk of bias. We used the ROBINS‐1 tool [15] to assess the risk of bias in nonrandomized studies by evaluating seven domains: confounding bias, bias arising from the selection of the participants, bias in the classification of the interventions, bias due to deviation from intended interventions, bias due to missing outcomes, bias in measurement of the outcomes, and bias in selection of the reported results. The reviewers resolved any conflicts by consensus.

### 2.6 Statistical Analysis

For binary outcomes, the results were reported as risk ratios (RR) along with their 95% confidence intervals (CIs). Pooled continuous endpoints were reported as mean differences (MDs) with 95% CIs. A random‐effects model was applied in all meta‐analyses. To evaluate heterogeneity among the included studies, the *I* ^2^ statistic was utilized. Heterogeneity was categorized as low, moderate, or high based on *I* ^2^ values of 25%, 50%, and 75%, respectively. In cases where significant heterogeneity was observed. Sensitivity analyses were performed to ensure the robustness of the results. This involved excluding studies with a high risk of bias or those identified as outliers. A subgroup analysis was performed according to the study design (RCTs vs. observational studies). The statistical analyses were conducted using Review Manager (RevMan), version 5.4, developed by The Cochrane Collaboration (2020).

## 3 Results

### 3.1 Search Results and Study Selection

**FIGURE 1:**
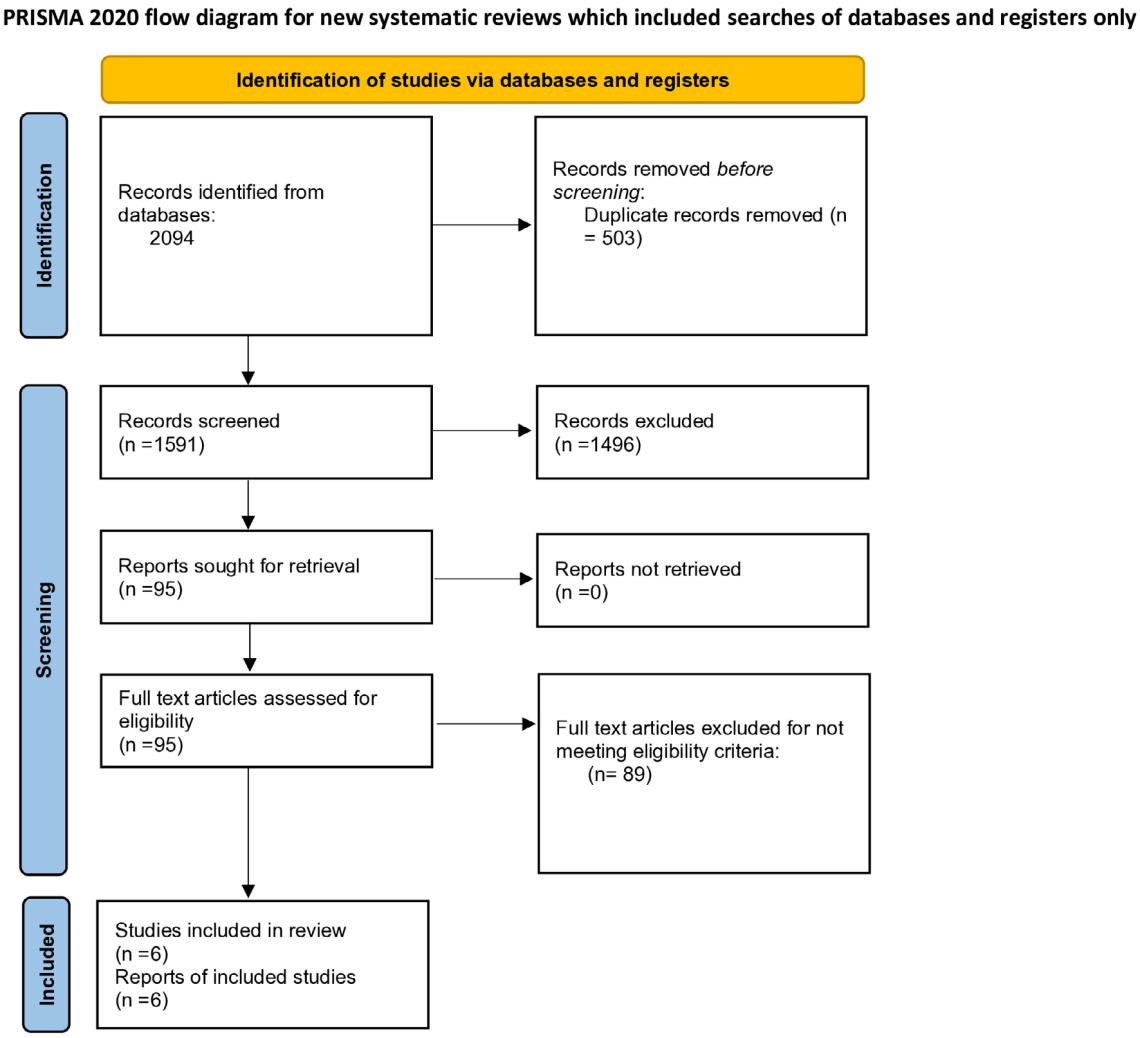
PRISMA flowchart illustrating the study selection process.

The literature search initially retrieved a total of 2094 records. After removing duplicates, 1591 unique citations were screened by title and abstract. Of these, 95 were selected for full text review based on relevance to the inclusion criteria. Following detailed assessment, 6 studies were deemed eligible and included in the final analysis [10, 11, 12, 16, 17, 18] (Figure 1).

### 3.2 Characteristics of Included Studies

A total of six studies met the eligibility criteria, including two RCTs and four observational studies. Collectively, these studies enrolled 1928 patients, 642 treated with PFA and 1286 with CTA (611 RFA and 675 CBA). The weighted mean follow‐up time was 10 months. A comprehensive overview of study characteristics and baseline patient demographics can be found in Tables 1 and 2.

**TABLE 1:** Study characteristics.

Study ID	Study design	Country	Number of patients in each group		Follow‐up period in months	Intervention	Control	Primary outcome
			Intervention	Control				
Cochet 2021 [16]	Prospective, single‐center, cohort study	France	18	23	3	Pulsed‐field ablation FARAPULSETM System (Boston Scientific)	RFA (16) and CBA (7)	Assess injury on the esophagus, descending aorta, and Phrenic nerve.
Nakatani 2021 [17]	Prospective, single‐center, cohort study	France	18	23	9	Pulsed‐field ablation FARAPULSETM System (Boston Scientific)	RFA (16) and CBA (7)	Compare the left atrial (LA) structural and mechanical characteristics
Maurhofer 2023 [18]	Prospective, single‐center, cohort study	Switzerland	40	160	12	Pulsed‐field ablation FARAPULSETM System (Boston Scientific)	RFA (80) and CBA (80)	Recurrence of any atrial tachyarrhythmia
Reddy 2023 [10]	Randomized controlled trial (RCT)	USA	305	302	12	Pulsed‐field ablation FARAPULSETM System (Boston Scientific)	RFA (167) and CBA (135)	Freedom from a composite of initial procedural failure, documented atrial tachyarrhythmia after a 3‐month blanking period, antiarrhythmic drug use, cardioversion, or repeat ablation.
Rocca 2024 [12]	Propensity score matched, Prospective, multi‐center, cohort study	Belgium, France, Italy	174	696	12	Pulsed‐field ablation FARAPULSETM System (Boston Scientific)	RFA (348) and CBA (348)	Freedom from any atrial tachyarrhythmia > 30 s off AADs and occurring after the 3‐month blanking period.
Reichlen 2025 [11]	Randomized controlled trial (RCT)	Switzerland	105	105	12	Pulsed‐field ablation FARAPULSETM System (Boston Scientific)	CBA	The first recurrence of any atrial tachyarrhythmia (atrial fibrillation, flutter, or tachycardia) lasting at least 30 s during the period from Day 91 to Day 365 after the ablation procedure.

Abbreviations: CBA, cryoballoon ablation; RFA, radiofrequency ablation.

**TABLE 2:** Baseline patient demographics.

Study ID	Age, mean (SD)		BMI, mean (SD)		CHA2DS2VAS, mean (SD)		LVEF, mean (SD)		LA diameter (mm), mean (SD)	
	Intervention	Control	Intervention	Control	Intervention	Control	Intervention	Control	Intervention	Control
Cochet 2021 [16]	58 (9)	59 (9)	26 (4)	26 (3)	0.5 (0.8)	0.7 (0.8)	62 (6)	61 (8)	NA	NA
Nakatani 2021 [17]	58 (9)	59 (9)	26 (4)	26 (3)	0.5 (0.8)	0.7 (0.8)	62 (6)	61 (8)	NA	NA
Maurhofer 2023 [18]	62.6 (9.6)	62.7 (12.1)	25.9 (4.1)	25.9 (3.7)	NA	NA	58.3 (3.8)	58.3 (3.8)	41.7 (5.4)	41 (7.2)
Reddy 2023 [10]	62.4 (8.7)	62.5 (8.5)	28.3 (4.6)	29.0 (4.8)	1.7 (1.2)	1.7 (1.2)	NA	NA	38.8 (5.7)	39.6 (5.8)
Rocca 2024 [12]	62 (11.6)	62.89 (12.36)	27 (4.8)	27.88 (5.07)	2 (1.48)	2 (1.48)	59.4 (4.2)	56.93 (8.70)	41.8 (4.9)	41.62 (4.50)
Reichlen 2025 [11]	64.0 (9.4)	63.3 (9.6)	27.0 (3.9)	27.3 (4.7)	2 (1.48)	2 (1.48)	60 (7)	60 (6)	39 (5)	38 (6)

Abbreviations: BMI, body mass index; CHA2DS2‐VASc, congestive heart failure, hypertension, age ≥ 75 years, diabetes mellitus, stroke or transient ischemic attack, vascular disease, age 65–74 years, and sex category (female); LVEF, left ventricular ejection fraction.

### 3.3 Quality Assessment and Risk of Bias

The two RCTs, Reddy et al. and Reichlen et al. [10, 11] showed low risk of bias according to the ROB‐2 tool. A visual summary of the risk of bias assessment is presented in Figure S1. The observational studies showed a low risk of bias according to the ROBINS I tool [12, 16, 18], except for Nakatani et al. [17], which showed some concerns regarding the measurement of the outcomes. The detailed assessment is shown in Figure S2.

### 3.4 Primary Outcome

**FIGURE 2:**
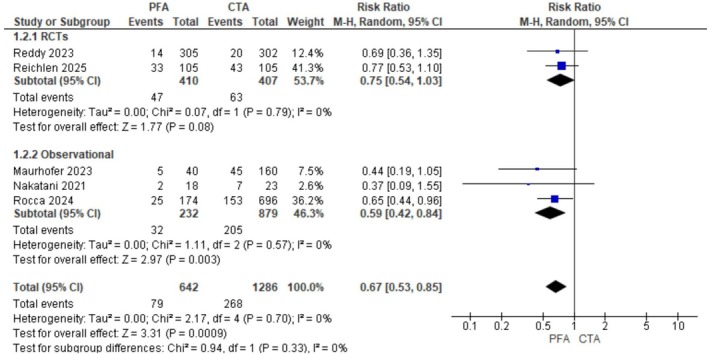
Forest plot depicting the comparison of atrial fibrillation (AF) recurrence between pulsed field ablation (PFA) and conventional thermal ablation (CTA). The plot shows the risk ratio (RR) with 95% confidence intervals (CIs) for each included study, along with the pooled estimate.

PFA was associated with a lower incidence of AF recurrence (RR: 0.67; 95% CI: 0.53–0.85; *p* = 0.0009, *I* ^2^ = 0%; Figure 2). The subgroup analysis revealed that this effect was mainly driven by observational studies (RR: 0.59; 95% CI: 0.42–0.84; *p* = 0.003, *I* ^2^ = 0%) and not RCTs (RR: 0.75; 95% CI: 0.54–1.03; *p* = 0.08, *I* ^2^ = 0%).

### 3.5 Secondary Outcomes

**FIGURE 3:**
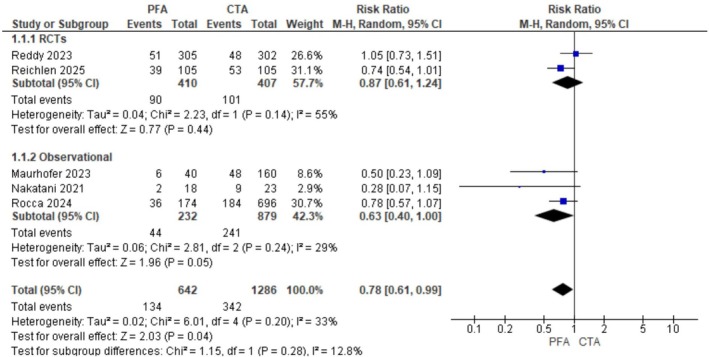
Forest plot depicting the comparison of any atrial arrhythmia recurrence between PFA and conventional thermal ablation (CTA). The plot shows the risk ratio (RR) with 95% confidence intervals (CIs) for each included study, along with the pooled estimate.

Regarding any atrial arrhythmia recurrence, the effect was less pronounced, favoring PFA (RR 0.78, 95% CI: 0.61–0.99; *p* = 0.04, *I* ^2^ = 33%; Figure 3). Sensitivity analysis was done to resolve heterogeneity and showed that PFA significantly reduced the incidence of any atrial arrhythmia recurrence after omitting Reddy et al. [10] (Figure S3A).

**FIGURE 4:**
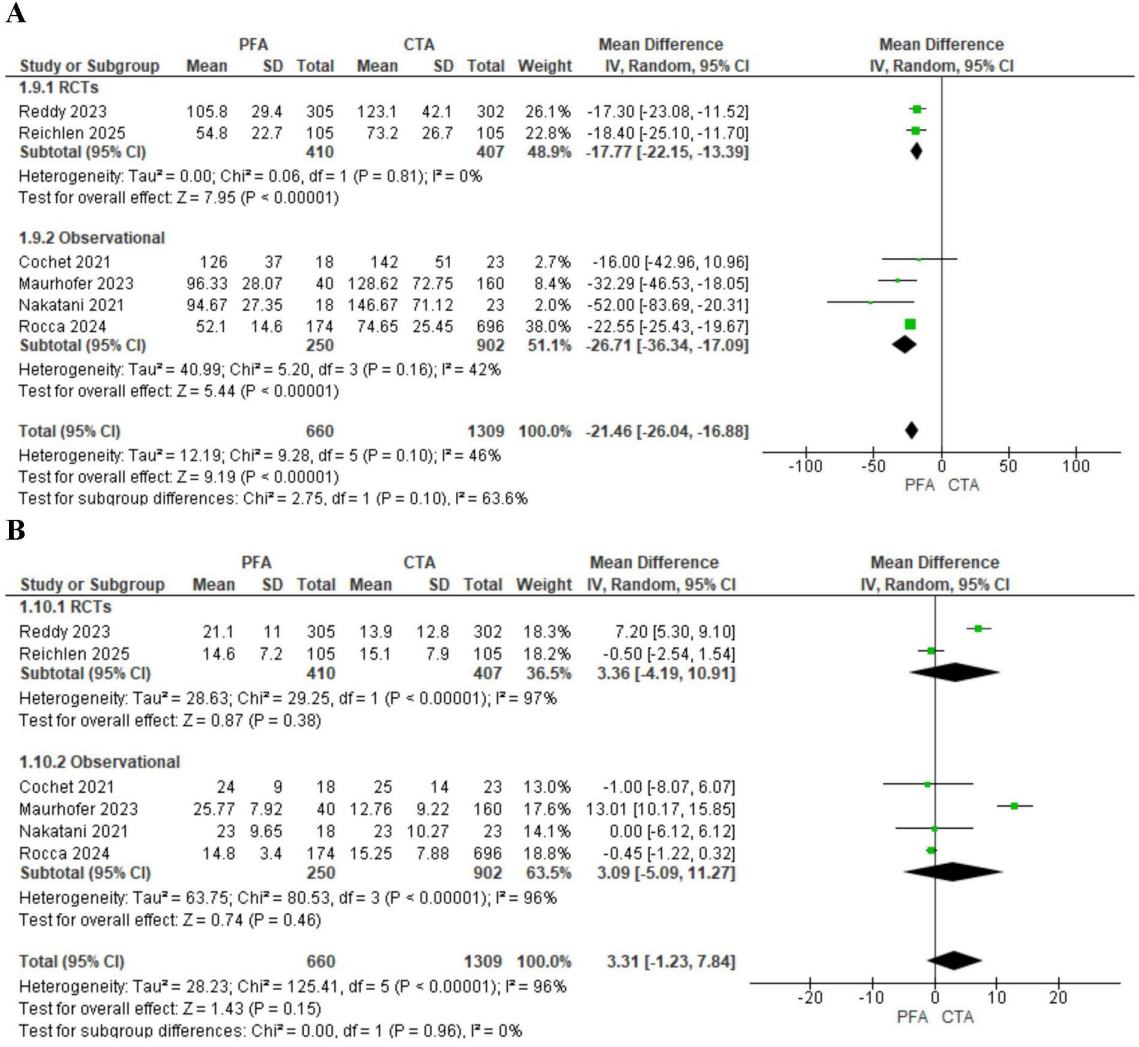
Forest plots comparing (A) total procedure time and (B) total fluoroscopy time between pulsed field ablation (PFA) and conventional thermal ablation (CTA). The plots show mean differences (MDs) with 95% confidence intervals (CIs).

The total procedure duration was significantly shorter with PFA (MD = −21.46 min, 95% CI: −26.04 to −16.88; *p* < 0.00001, *I* ^2^ = 46%, Figure 4A). Fluoroscopy time was not different between both groups (MD = −3.31 min; 95% CI: −1.23 to 7.84; *p* = 0.15, Figure 4B). However, heterogeneity for this outcome was substantial (*I* ^2^ = 96%, *p* < 0.00001). Sensitivity analysis was used to resolve heterogeneity, and the results remained consistent (Figure S3B).

Regarding safety outcomes, esophageal lesions were reported exclusively in patients undergoing CTA, with 10 events in the CTA group and none in the PFA group. Phrenic nerve palsy occurred less frequently with PFA (4 events) compared to CTA (25 events). However, since the event rates were very low and the data were limited to only two studies, a meta‐analysis for these outcomes was not conducted.

**FIGURE 5:**
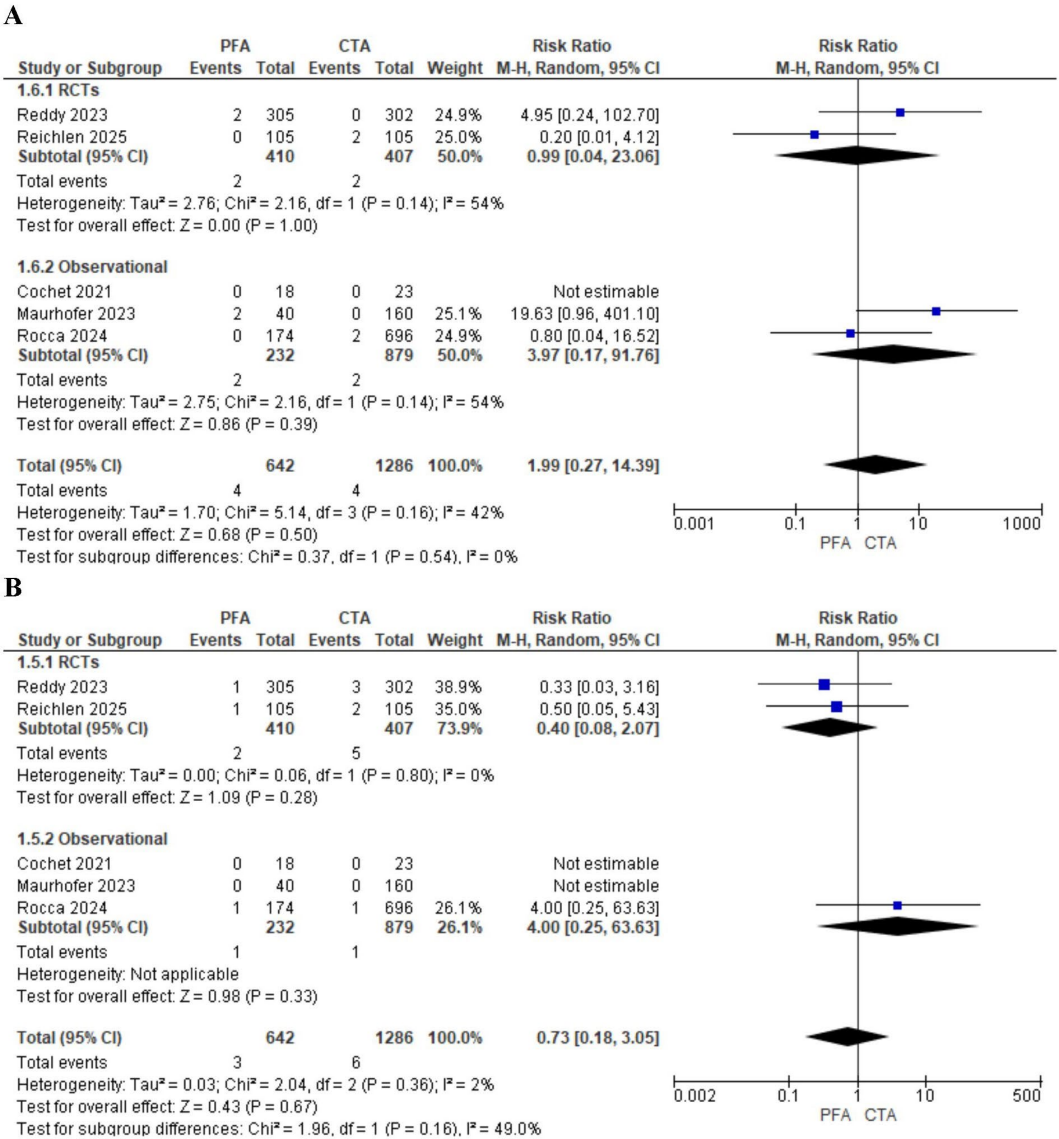
Forest plots comparing the incidence of (A) pericardial tamponade and (B) stroke or transient ischemic attack (TIA) between pulsed field ablation (PFA) and conventional thermal ablation (CTA). The plots display risk ratios (RR) with 95% confidence intervals (CIs).

There was no difference in the incidence of pericardial tamponade (OR: 1.85; 95% CI: 0.59–5.80; *p* = 0.29, *I* ^2^ = 42%; Figure 5A) and stroke or TIA (OR: 0.66; 95% CI: 0.17–2.58; *p* = 0.55, *I* ^2^ = 2%; Figure 5B). Sensitivity analysis was done to resolve heterogeneity in the pericardial effusion outcome, and the estimated effect remained non‐significant (Figure S3C).

## 4 Discussion

In this meta‐analysis of six studies (two RCTs and four observational studies) with 1928 patients, we demonstrated that PFA was associated with a lower incidence of AF or any atrial arrhythmia recurrence. PFA was also associated with a significantly shorter procedure duration, without increasing fluoroscopy time or the incidence of cardiac tamponade and stroke/TIA.

We demonstrated that PFA was associated with a lower incidence of recurrence, whether it was the recurrence of AF or any other type of arrhythmia. However, it is important to note that the overall benefit was primarily driven by the observational studies rather than RCTs, which are prone to selection and confounding biases. Previous meta‐analyses investigating the efficacy of PFA have shown inconsistent results regarding the recurrence of AF and any atrial arrhythmia. In addition, they generally investigated the efficacy of PFA across both paroxysmal and persistent AF without stratification by subgroup, which might have caused some inconsistency in the outcomes [19, 20, 21, 22]. Furthermore, our pooled analysis showed that the efficacy of PFA was maintained over a 1‐year follow‐up period. This aligns with the previously published reports, which demonstrated that an arrhythmia‐free survival period achieved with PFA could last up to 49 months of follow‐up [23]. It's important to note that none of the included studies reported performing ablation beyond PVI, except for Reichlen et al. [11], which reported that cavotricuspid isthmus (CTI) RFA was performed in 14 patients in the PFA group and 12 patients in the CBA group. This ablation may decrease the recurrence of atrial arrhythmias, especially atrial flutter [24]. Nevertheless, there was no significant difference in its use between the two groups, which makes it unlikely to have affected the results of the meta‐analysis.

Another potential advantage of PFA over CTA is the shorter required total procedure time, which is confirmed by our analysis. This is mainly attributed to the fact that PFA can rapidly isolate all four pulmonary veins within just a few applications, significantly reducing the total procedure time [25]. On the other hand, our pooled results demonstrated that the duration required to perform adequate fluoroscopy did not differ between PFA and CTA, which is consistent with previous meta‐analyses [19, 21], while the studies conducted by Campos et al. and Aldaas et al. reported longer fluoroscopy time with PFA [20, 22]. The extensive usage of non‐fluoroscopic electro‐anatomical mapping technologies with thermal ablation and possible lack of experience by operators may explain this problem. When mapping systems are integrated and expertise with PFA grows, it is expected that the time spent using fluoroscopy might decrease [26].

A theoretical advantage for PFA over CTA is the non‐selectivity of thermal ablation techniques, which results in damage to the nearby structures, such as the esophagus and the phrenic nerve [20]. Additionally, this excessive damage can provoke inflammation and lead to the formation of a heterogeneous scar lesion that itself can induce the formation of new arrhythmias. It is important to note that PFA has been associated with a reduced risk of atrio‐esophageal injury and phrenic nerve palsy, representing a significant safety advantage over other ablation modalities [27, 28, 29]. Nevertheless, it should be noted that in our meta‐analysis, esophageal injury was only reported in one study, and the lower incidence of phrenic nerve palsy was only reported in two studies, with too low an event rate to meta‐analyze these two outcomes.

Two studies have demonstrated that patients who underwent PFA were associated with a higher incidence of cardiac perforation or tamponade compared to patients who received thermal ablation [20, 21]. Our pooled findings contradict the findings of the previous study, as we observed no significant difference between the two techniques. This can be explained by the relatively small number in our meta‐analysis compared to the previous ones, as this meta‐analysis exclusively focuses on paroxysmal AF patients. This complication could be explained by the relative inexperience of the practitioner with the PFA devices, as this technology is still relatively new and operators may not have the familiarity and nuanced understanding necessary to minimize risks effectively [30]. With more experience and refined techniques, it is expected that the incidence of such complications will be reduced. Additional safety analysis revealed that the risk of stroke/TIA was rare and comparable between the two techniques, which is consistent with previous findings, confirming the safety profile of the ablation technique.

Several ongoing trials are expected to generate further evidence regarding the efficacy and safety of PFA in paroxysmal AF [31, 32, 33]. The “inspIRE” trial has been completed, with its results currently available. Their primary safety outcome was the occurrence of procedure‐related adverse events, including atrio‐esophageal fistula and cardiac tamponade, on which they reported zero events as well, while their efficacy outcomes include freedom from different types of atrial arrhythmia [31]. The results of this trial, along with those from other studies, are anticipated to strengthen the current evidence. However, it's also important to note that new strategies and new devices, including VARIPULSE (Johnson and Johnson) and PulseSelect (Medtronic), are being used to deliver the pulsed field ablation (PFA) in these trials. Further meta‐analyses that incorporate these upcoming trials will be important for evaluating the differential effects of various PFA devices, allowing for a comprehensive understanding of their comparative efficacy and safety profiles.

Our focus on paroxysmal AF represents a key strength, especially in light of recent meta‐analyses that addressed PFA in AF overall (both paroxysmal and persistent combined). By specifically evaluating paroxysmal AF, we aimed to obtain more precise results for this distinct patient subgroup. Our approach was inspired by two recent RCTs included in our study, Reddy et al. and Reichlen et al. [10, 11], both of which focused exclusively on paroxysmal AF. This study has some limitations that should be considered. Most of the included studies were observational, which might introduce selection bias and unmeasured confounding. Additionally, the estimated effects in the recurrence outcomes were primarily driven by the observational studies, which makes the results of these outcomes less robust. Heterogeneity was observed in our results, showing that operator experience, patient groups, tools, and hospital practices differed between trials. Since centers used different pulse settings, catheters, and strategies for lesions, we could not account for these differences in the analysis. Moreover, we could not comment on the risk of long‐term recurrence. It is also important to note that all of the included studies used the FARAPULSE system, which may make our findings not generalizable to other PFA systems currently under investigation, such as the VARIPULSE and PulseSelect. Finally, this meta‐analysis lacks patient‐level data that precludes granular analysis for the subgroups who might drive the most benefit from PFA.

## 5 Conclusions

Among patients with paroxysmal AF undergoing catheter‐based interventions, PFA was associated with a lower incidence of recurrence, shorter procedures, and did not increase the risk of complications. However, given the limited number of RCTs, the fact that the significance of the recurrence outcomes was more influenced by observational studies than by the RCTs, and the short follow‐up period, additional RCTs with longer follow‐up are necessary to confirm these findings.

## Author Contributions

A.E.M., A.F.G., Z.K., and M.E. wrote the main manuscript. A.E.M. and M.E. prepared the figures. A.D., R.R., A.A., I.T., M.E., R.A., E.M., and H.A.M.D. extracted the data and prepared the tables. A.E., J.Y., and I.Y.E. reviewed the manuscript.

## Ethics Statement

The authors have nothing to report.

## Consent

All authors approved the final manuscript and the submission to this journal.

## Conflicts of Interest

The authors declare no conflicts of interest.

## Supporting information


**Table S1:** PRISMA guidelines checklist.
**Table S2:** Detailed search strategy.
**Figure S1:** ROB2 quality assessment.
**Figure S2:** ROBINS I quality assessment.
**Figure S3A:** All atrial arrhythmia recurrence after sensitivity analysis.
**Figure S3B:** Total procedure time after sensitivity analysis.
**Figure S3C:** Pericardial tamponade after sensitivity analysis.
